# Effects of Seasonal Conditions on Abundance of Malaria Vector *Anopheles stephensi* Mosquitoes, Djibouti, 2018–2021

**DOI:** 10.3201/eid2904.220549

**Published:** 2023-04

**Authors:** Alia Zayed, Manal Moustafa, Reham Tageldin, James F. Harwood

**Affiliations:** US Naval Medical Research Unit No. 3, Cairo Detachment, Cairo, Egypt (A. Zayed, M. Moustafa, R. Tageldin);; US Naval Medical Research Unit No. 3, Sigonella, Italy (J. Harwood); Cairo University, Cairo, Egypt (A. Zayed)

**Keywords:** malaria, vector-borne infections, *Anopheles stephensi*, parasites, distribution, environmental effects, seasonality, temperature, rainfall, Djibouti

## Abstract

We describe the influence of seasonal meteorologic variations and rainfall events on *Anopheles stephensi* mosquito populations during a 40-month surveillance study at a US military base in Djibouti. Focusing surveillance and risk mitigation for *An. stephensi* mosquitoes when climatic conditions are optimal presents an opportunity for malaria prevention and control in eastern Africa.

*Anopheles stephensi* mosquitoes, an urban malaria vector, have established robust populations in the Horn of Africa. Since the mosquito’s detection in 2012 (*1*), malaria cases in Djibouti increased 42.9-fold during 2013–2021, reaching ≈72,300 cases (*2*). Before introduction of *An. stephensi* mosquitoes, Djibouti was approaching the preelimination phase for malaria (*3*). Because *An. stephensi* mosquitoes are competent vectors for *Plasmodium falciparum* and *P. vivax* parasites (*3*), WHO considers this mosquito species a major threat to malaria elimination in Africa (*4*). *An. stephensi* mosquitoes have also been detected in Sudan, Ethiopia, and Somalia (*5**–**8*). Understanding *An. stephensi* mosquito adaptation to environmental conditions affecting population dynamics in urban settings is crucial in Africa. *An. stephensi* mosquitoes abundance (number of mosquitoes collected per trap night) changed from seasonal during fall–spring 2013–2016 to year-round in 2017 (*3*). Since *An. stephensi* mosquitoes were introduced, malaria cases have increased among military personnel, some immunologically naive, deployed as members of multinational militaries in Djibouti (*9*). Camp Lemonnier (CLDJ), a US naval base, has urban characteristics similar to the city of Djibouti, in which it is located. For this study, we monitored vector dynamics on the base, providing data to help inform health protection strategies among both military and civilian populations. 

## The Study

In coordination with the CLDJ Expeditionary Medical Facility, during January 2018–April 2021, we conducted weekly mosquito surveillance at 32 on-base sites covering 2 km^2^ and stored information in dataset A. In October 2019, we began identifying monthly captures of *An. stephensi* mosquitoes specifically (i.e., identified at the species level) (dataset B). We set US Centers for Disease Control and Prevention (CDC) CO_2_-baited Miniature Light traps (https://www.cdc.gov/mosquitoes/guidelines/west-nile/surveillance/environmental-surveillance.html) and Woodstream Mosquito Magnet (MM) propane-generated CO_2_ traps (https://www.woodstream.com) overnight near dwellings, dining areas, sport facilities, and other areas frequented by humans. We identified *Anopheles* species on the basis of criteria published elsewhere (*10*,*11*). We analyzed abundance data in the context of specific weather events and seasonal climatic trends at the time of collection. We obtained meteorologic data from several sources (Appendix), using latitude 11.54733 N and longitude 43.15948 E (0.6–1.2 km from study sites) for location and a locally appropriate meteorologic calendar to determine seasons. We assessed the effects on *An. stephensi* mosquito abundance of monthly mean temperatures and rainfall amounts at time of precipitation and at 2-week, and 1- and 2-month lag times (i.e., time after rainfall). We did not consider longer lag times because of the likely effects of evaporation. 

We used the Shapiro-Wilk test to check normal distribution of *An. stephensi* mosquito data and Pearson correlation coefficient to evaluate relationships between mosquito abundance and climatic variables. We categorized temperatures as either above or equal to or below median annual temperature (30°C). We grouped rainfall data according to frequency at each of 5 levels: 0, 0.2–4.9, 5–21, 21.1–39.9, and 40–155 mm/week. We used Poisson regression for univariate and multivariate analyses to determine associations between mosquito abundance and predictor variables, and used PROC GENMOD in SAS version 9.4 (SAS Institute, Inc., https://www.sas.com) to perform logistic regression. We expressed results in incidence rate ratios (IRR) and used p = 0.05 as the cutoff for statistical significance. 

**Figure 1:**
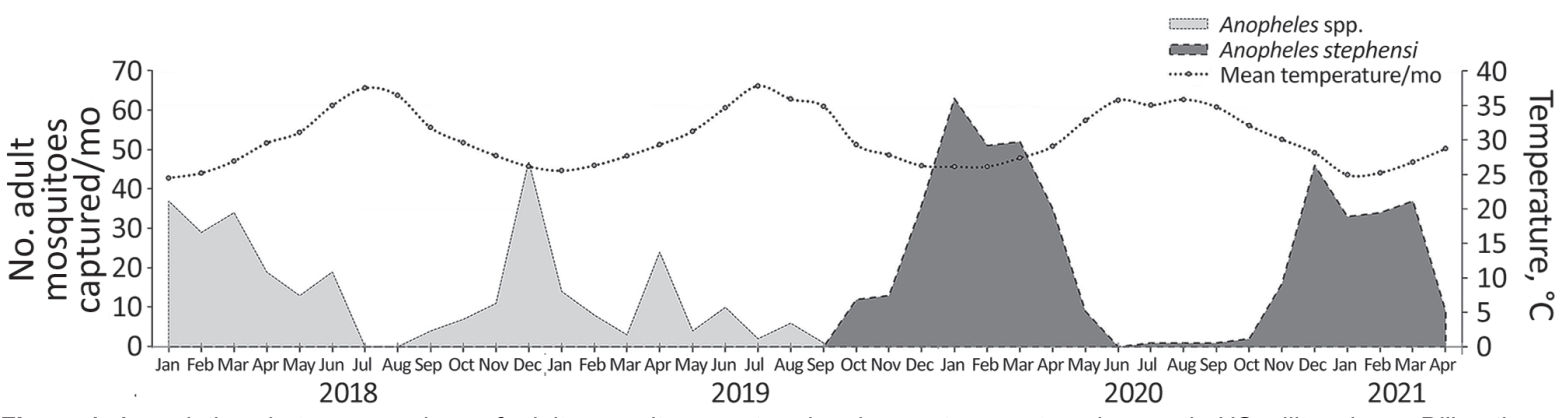
Associations between numbers of adult mosquitoes captured and mean temperature, by month, US military base, Djibouti, September 2019–August 2020. We began identifying *Anopheles stephensi* mosquitoes specifically in October 2019.

*An. stephensi* represented 95.6% of all *Anopheles* spp. mosquitoes we identified. Using dataset B to compare effectiveness of trap types, we found that MM traps captured 25.6% more *An. stephensi* mosquitoes than did CDC traps (IRR 2.3; p<0.0001) (Appendix Table, Figure). Univariate regression analysis of datasets A and B (Appendix Table) demonstrated that *An. stephensi* mosquito populations persisted year-round. Related to seasonal distribution, in dataset A, winter accounted for 56.4% of *Anopheles* spp. mosquito captures; spring, 28.1%; fall, 9.8%; and summer, 5.7%. In dataset B, winter accounted for 55.2% of *An. stephensi* mosquito captures; spring, 37.1%; fall, 6.9%; and summer, 0.8%*.* Associations between *An. stephensi* mosquito abundance and monthly mean temperatures (Figure 1) were positive for temperatures <30 (IRR 5.5 for dataset A, 7.4 for dataset B; p<0.0001). In dataset A, 85% of *Anopheles* spp. mosquitoes were collected at temperatures ≤30°C; for dataset B, the percentage was 94% of *An. stephensi* mosquitoes (Appendix Table). 

**Figure 2:**
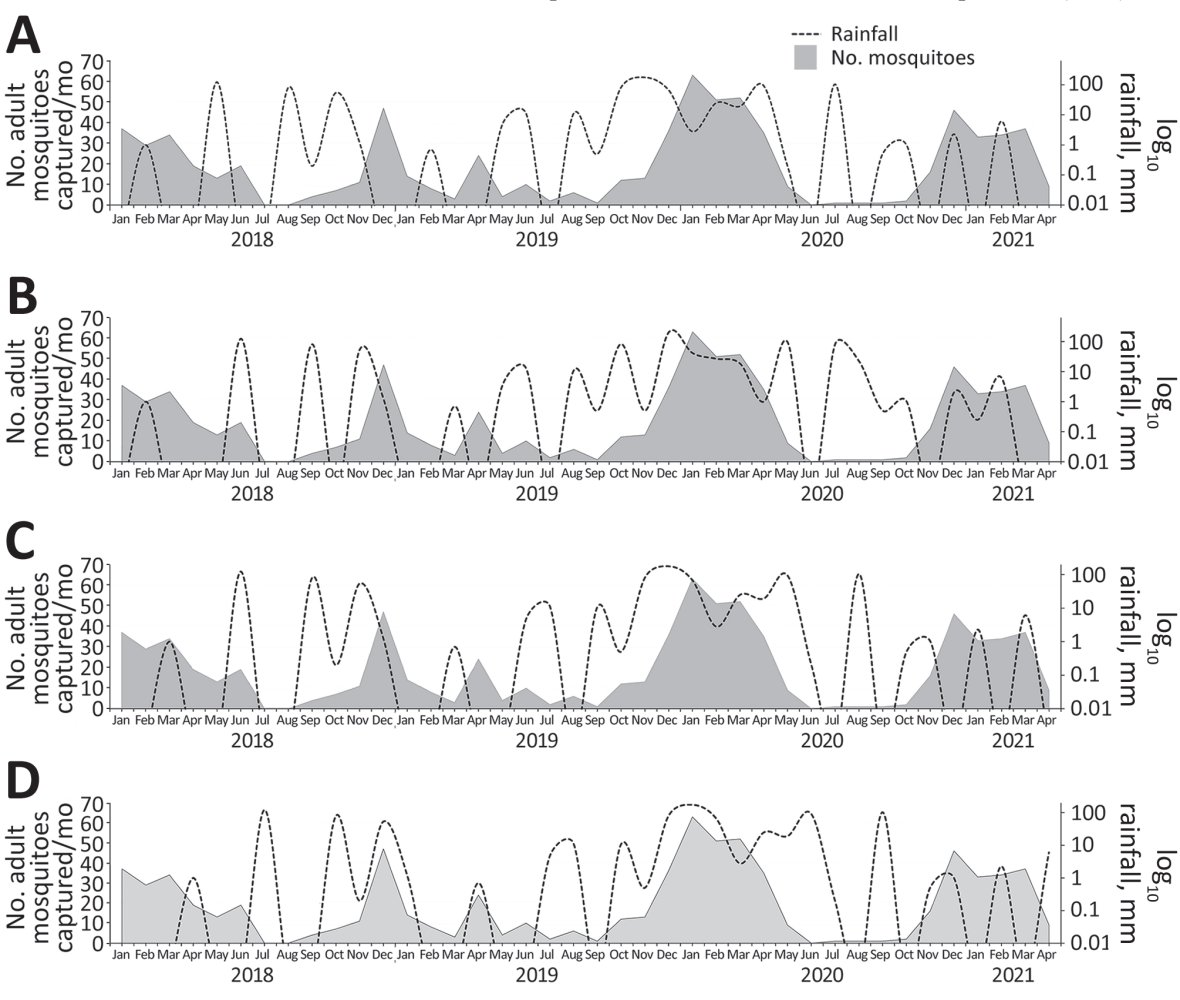
Associations between monthly collected numbers of *Anopheles stephensi* mosquitoes captured and precipitation rates, US military base, Djibouti, September 2019–August 2020. A) At time of rainfall; B) 2 weeks after rainfall; C) 1 month after rainfall; D) 2 months after rainfall.

Mosquito abundance increased 4–8 weeks after flooding in November 2019 (Figure 2). We also analyzed data on mosquito abundance 2 weeks and 1 and 2 months after rainfall throughout September 2019–August 2020, during which time 2 floods occurred (Table 1). Regression analysis showed significant associations between rainfall and *Anopheles* mosquito abundance recorded 2 weeks (IRR 2.4), 1 month (IRR 2.99), and 2 months (IRR 2.75) after periods of rainfall 21.1–39.9 mm/week (p<0.0001), corresponding to average mosquito counts of 9.6 (2 weeks), 11.3 (1 month), and 11.0 (2 months) after the rainfall. Unexpectedly, mosquito abundance also increased significantly 2 weeks (IRR 2.59), 1 month (IRR 2.58), and 2 months (IRR 2.00; p <0.0001) after periods of rainfall of just 0.2–4.9 mm/week. Multivariate analysis indicated that season and temperature were the variables most significantly associated with mosquito abundance when analyzed with no lag or 1-month rainfall lag effect. Winter (IRR 4.2 [no lag], 4.1 [1-month lag]; p<0.0001) and spring (IRR 2.8 [no lag], 2.9 [1-month lag]; p<0.0001) were the factors most associated with increases in *Anopheles* mosquitoes, followed by temperatures ≤30°C (IRR 2.4 [no lag], 2.2 [1-month lag]; p<0.0001) (Table 2). 

**Table 1:** Univariate Poisson regression analysis of lagged effects of rainfall on abundance of *Anopheles stephensi* mosquitoes 2 weeks, 1 month, and 2 months after rainfall periods, US military base, Djibouti, September 2019–August 2020*

Time after rainfall	Rainfall level, mm/wk	Regression analysis		Abundance
		IRR (95% CI)	p value	
2 wk	40–155	0.56 (0.3–1.1)	0.09	2.3
	21.1–39.9	2.4 (1.7–3.4)	<0.0001	9.6
	5–21	1.5 (0.9–2.5)	0.11	6
	0.2–4.9	2.59 (2–3.4)	<0.0001	10.4
	0	Referent		4
1 mo	40–155	1.86 (0.9–2.2)	0.009	7
	21.1– 39.9	2.99 (2–3.8)	<0.0001	11.3
	5–21	1.13 (0.9–2.4)	0.6	4.3
	0.2–4.9	2.58 (1.5–2.7)	<0.0001	9.8
	0	Referent		3.8
2 mo	40–155	1.37 (1.2–3)	0.17	5.5
	21.1–39.9	2.75 (2.1–4.2)	<0.0001	11
	5–21	1.42 (0.7–1.9)	0.18	5.7
	0.2–4.9	2 (1.9–3.5)	<0.0001	8
	0	Referent		4

*Abundance is the average number of mosquitoes per trap night. IRR, incidence rate ratio.

**Table 2:** Multivariate Poisson regression analysis of seasonal and climatic factors associated with *Anopheles stephensi* mosquito abundance with and without lag effect after rainfall periods, US military base, Djibouti, September 2019–August 2020*

Variable	No lag effect			1-mo lag effect	
	IRR (95% CI)	p value		IRR (95% CI)	p value
Seasons					
Winter	4.2 (2.7–6.3)	<0.0001		4.12 (2.7–6.2)	<0.0001
Spring	2.8 (1.9–4.2)	<0.0001		2.86 (1.9–4.2)	<0.0001
Fall	1.3 (0.8–1.9)	0.3		1.19 (0.8–1.8)	0.42
Summer	Referent			Referent	
Temperature, °C					
<30	2.4 (1.9–3.1)	<0.0001		2.2 (1.7–2.9)	<0.0001
>30	Referent			Referent	NA
Rain amounts, mm/wk					
40–155	0.33 (0.2–0.7)	0.004		1.2 (0.8–1.8)	0.4
21.1–39.9	1.1 (0.8–1.5)	0.6		1.5 (1.2–2.1)	0.0024
5–21	0.9 (0.6–1.3)	0.53		0.9 (0.6–1.5)	0.7
0.2–4.9	0.7 (0.6–0.9)	0.005		1.4 (1.2–1.7)	0.0002
0	Referent			Referent	
*NA, not applicable; IRR, incidence rate ratio.					

## Conclusions 

We speculate that the slow continuous release of CO_2_ of MM traps contributed to higher captures of *An. stephensi* mosquitoes than for CDC traps. In a study in Malaysia, MM traps performed 3-fold better than CDC traps for capturing *Anopheles* spp. mosquitoes (*12*), demonstrating the suitability of MM traps for *An. stephensi* mosquito surveillance in urban settings and areas with limited or no access to dry ice (*13*). 

*An. stephensi* mosquitoes were present year-round but at substantially higher populations during winter (mean temperature 26°C, average rainfall 2.3 mm/week) and spring (mean temperature 29°C, average rainfall 7.3 mm/week). A previous study observed a similar link between temperature and *An. stephensi* mosquito populations, with 29°C assessed as optimal (*14*). We linked the bionomics of *An. stephensi* mosquito abundance in urban areas to human-modified conditions, such as air conditioning–produced condensation, water storage tanks, open jerry cans, and water-filled tires following rainfall, all of which increased favorable mosquito habitats (*1*) and in which we observed larval habitats around CLDJ. Flash flooding in Djibouti did not increase *An. stephensi* mosquito abundance. In fact, flooding might have destroyed laid eggs, hatched larvae, and temporary larval habitats, as was reported in China (*15*), possibly explaining higher population growth after periods of rainfall of 21.1–39.9 mm/week than 40–155 mm/week. Because breeding sites in urban areas depend as much on human-generated water sources as rainfall, adult mosquitoes were able to persist even during periods of low precipitation (*14*). We found that periods of rainfall at 21.1–39.9 mm/week and temperatures slightly <30°C were optimal for adult *An. stephensi* mosquito abundance. Therefore, surveillance and control efforts should be most intense during times of the year when these conditions are common. However, because *An. stephensi* mosquitoes are present year-round, prevention and control measures cannot be relaxed during any season (Appendix). 

Although our study was set at CLDJ facilities, conditions were comparable to other urban settings in Djibouti, which should encourage local health authorities to benefit from our data. The persistence of mosquito populations at CLDJ, which regularly monitors and employs control efforts, should raise the alarm for increased malaria risk in densely populated city areas with fewer public health and disease control resources. Given limited resources, we recommend targeted reduction of *An. stephensi* larval habitat in this area. 

Appendix Additional information about effects of seasonal conditions on abundance of malaria vector *Anopheles stephensi*, Djibouti, September 2019–August 2020.
