# Supplementary material for: Effects of Seasonal Conditions on Abundance of Malaria Vector Anopheles stephensi Mosquitoes, Djibouti, 2018–2021
Source: Emerg Infect Dis. 2023 Apr;29(4):801–5. doi: 10.3201/eid2904.220549 (PMC10045708; doi:10.3201/eid2904.220549)
Supplement: Appendix — Additional information about effects of seasonal conditions on abundance of malaria vector Anopheles stephensi, Djibouti, September 2019–August 2020. [file 22-0549-Techapp-s1.pdf]

Article DOI: https://doi.org/10.3201/eid2904.220549

Effects of Seasonal Conditions on Abundance of Malaria Vector *Anopheles stephensi* Mosquitoes, Djibouti City, 2018–2021

Appendix

Additional Methods

Mosquito Magnet

Operational manual: https://www.mosquitomagnet.com

Meteorological Records

Daily records were acquired from US Air Force 14th Weather Squadron, (https://climate.af.mil/product_locator/station_page?networktype=AMIL&platformid=KQRH;) with Tuitempo archived weather supplementing missing events, (https://en.tutiempo.net/climate/10-2019/ws-631252.html) and flash flooding data acquired from the United Nations' Reliefweb site (https://reliefweb.int/sites/reliefweb.int/files/resources/Joint%20Flash%20Report%20Djibouti%20Flood%20-%20Nov%202019.pdf).

Results

Univariate analysis of abundance of *Anopheles stephensi* corresponding to predictor variables, and the efficiency of collection methods:

Univariate regression analysis of datasets A and B records (Table 1) proved the abundance of *An. stephensi* in all seasons with lower numbers in summer. Winter scored the highest records among seasons representing 56.4% of total mosquito numbers of dataset A and 55.2% of B. Compared to numbers in summer, density of vector in A was 9.7-fold higher in winter ($P<0.0001$), 4.9-fold higher in spring ($P<0.0001$) and 1.7-fold higher in fall ($P=0.01$),

referring to IRR and parameter estimate values of the regression analysis. Number of mosquitoes in B was 52-fold higher in winter (P<0.0001), 36.6-fold higher in spring (P<0.0001) and 8.6-fold higher in fall (P=0.003) compared to summer records.

Trap Type Comparison

Comparison of the two trap types was conducted on surveillance data from October 2019 to April 2021 (Fig. 1).

Conclusion

The optimum temperature for *An. stephensi* adult population falls within the thermal breadth for transmission of *Plasmodium falciparum* and *P. vivax* (15.3-37.2 C and 15.7-32.5 C, respectively). This further exacerbates the year-round malaria transmission risk in Djibouti, knowing that *An.stephensi* is currently present in all seasons (*1*).

Reference

1. Villena OC, Ryan SJ, Murdock CC, Johnson LR. Temperature impacts the environmental suitability for malaria transmission by *Anopheles gambiae* and *Anopheles stephensi*. Ecology. 2022;103:e3685.

Appendix Table. Univariate analysis of abundance of *Anopheles stephensi* corresponding to predictor variables, and the efficiency of collection methods

	Regression analysis (A)*			Regression analysis (B)*		
Variables/ Methods	IRR (95%CI)	P	n (%)†	IRR (95%CI)	P	n (%)†
Seasons†						
Winter	9.7 (6.9, 13.4)	<0.0001	387 (56.4)	52 (13, 210)	<0.0001	143 (55.2)
Spring	4.9 (3.4, 6.8)	<0.0001	193 (28.1)	36.6 (9, 148.3)	<0.0001	96 (37.1)
Fall	1.7 (1.1, 2.5)	0.01	67 (9.8)	8.9 (2.1, 38.3)	0.003	18 (6.9)
Summer	Reference (1)		39 (5.7)	Reference (1)		2 (0.8)
Temperature, °C						
<u><</u>30	5.5 (4.5,6.6)	<0.0001	722 (85.1)	7.4 (5.0, 11.0)	<0.0001	407 (94)
>30	Reference (1)		126 (14.6)	Reference (1)		24 (6)
Trap type§						
MM				2.3 (1.8, 2.7)	<0.0001	258 (62.8)
CDC				Reference (1)		153 (37.2)

*Univariate Poisson regression analysis of data of the genus Anopheles from January 2018-April 2021 (Regression analysis (A)) and the precisely identified An. stephensi data from October 2019-April 2021 (Regression analysis (B)); CI: confidence intervals. IRR: Incidence Rate Ratio.
†Total number of mosquitoes with percentage of the collected mosquitoes represented for the variable in parentheses.
‡Seasonal analysis was conducted on data representing the four seasons of dataset A (March 2018-Febraury 2021) and the four seasons of dataset B (December 2019-November 2020).
§Trap types: MM: Mosquito Magnet. CDC: Disease Control and Prevention miniature light traps. Winter (December; January; February), spring (March; April; May), summer (June; July; August) and fall (September; October; November).

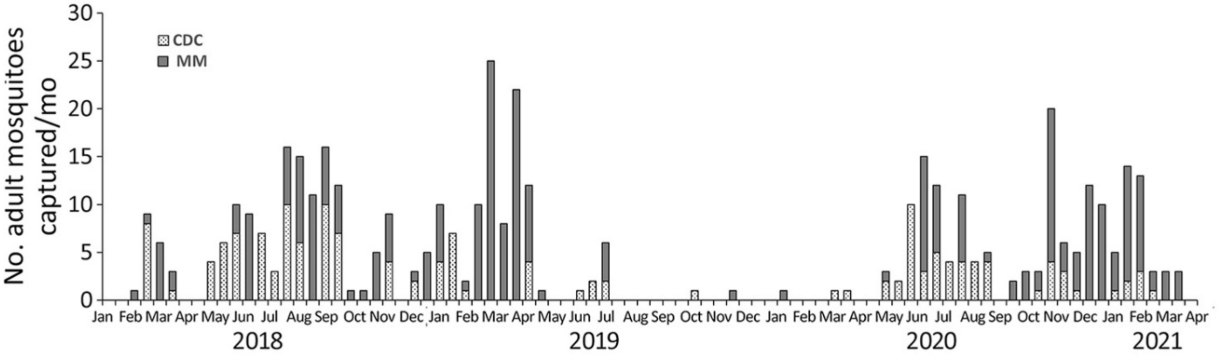

Appendix Figure. Weekly numbers of collected *Anopheles stephensi* from October 2019 to April 2021 using CDC or Mosquito Magnet (MM) traps.
